# Cascaded compressed-sensing single-pixel camera for high-dimensional optical imaging

**DOI:** 10.21203/rs.3.rs-2295079/v1

**Published:** 2023-01-09

**Authors:** Jongchan Park, Liang Gao

**Affiliations:** Department of Bioengineering, University of California, Los Angeles, CA, USA; Department of Bioengineering, University of California, Los Angeles, CA, USA

## Abstract

Single-pixel detectors are popular devices in optical sciences because of their fast temporal response, high sensitivity, and low cost. However, when being used for imaging, they face a fundamental challenge in acquiring high-dimensional information of an optical field because they are essentially zero-dimensional sensors and measure only the light intensity. To address this problem, we developed a cascaded compressed-sensing single-pixel camera, which decomposes the measurement into multiple stages, sequentially reducing the dimensionality of the data from a high-dimensional space to zero dimension. This measurement scheme allows us to exploit the compressibility of a natural scene in multiple domains, leading to highly efficient data acquisition. We demonstrated our method in several demanding applications, including enabling tunable single-pixel full-waveform hyperspectral light detection and ranging (LIDAR) for the first time.

## Introduction

Although century-old, single-pixel detectors are still prevalent in optical sciences because of their fast temporal response, high sensitivity over a broad spectrum, low dark noise, and low cost^1,2^. However, when being used for imaging, they are fundamentally limited by a “*dimensionality gap*”—while an optical field is characterized by a multidimensional plenoptic function^3^ with rich information, the single-pixel detector measures only the light intensity, leading to a zero-dimensional (0D) image.

The convergence of recent advances in spatial light modulating technologies^4^ and computational imaging shows great promise in bridging this gap. The resultant “single-pixel camera” has been demonstrated in myriad imaging applications^1,2, 5–12^. Moreover, using compressed sensing in single-pixel cameras^6,13^ further allows capturing light information with a sampling rate much lower than the Nyquist condition, leading to a more efficient data acquisition than conventional pixel-array cameras. The reduced sampling rate is particularly crucial for high-dimensional (high-D) imaging systems that inherently carry a heavy dataload. Nonetheless, the adoption of compressed single-pixel cameras in multidimensional optical imaging is hindered by two significant technical challenges: the choice of an appropriate compressed sampling scheme and the optical transformation of the data from a high-D space to a low-dimensional (low-D) space.

On the one hand, when choosing an appropriate sampling scheme for compressed imaging, the most common choice is to use a Hadamard basis. The Hadamard basis measurement, derived from a Hadamard matrix^14^ consisting of elements of + 1 and − 1, can be readily implemented in an optical system with a digital micromirror device (DMD)^15,16^, which modulates the light in a binary manner. A typical DMD-based compressed imaging system encodes the scene with a sequence of Hadamard patterns and measures the integrated signals using a single-pixel detector at each pattern. The image can then be reconstructed by correlating the encoding patterns with the signals obtained. However, when capturing an image at a sub-Nyquist sampling rate, that is, the image’s pixel count is much greater than the number of acquired samples, choosing an appropriate subset of the Hadamard basis becomes critical as it is often object-dependent^17,18^. Although regularization algorithms^19,20^ may remedy the result, they are ineffective in processing highly compressed measurements^21^.

On the other hand, capturing optical information beyond a two-dimensional (2D) intensity map—such as in hyperspectral^22^ or volumetric imaging^3^—with a single-pixel detector is nontrivial because most current spatial light modulators are in a 2D format. For multidimensional optical imaging, a high-D optical field must be first transformed to a low-D space so it can be modulated by a spatial light modulator. Such an operation is typically performed through temporal multiplexing. For example, by coupling a Fourier transform imaging spectrometer to a single-pixel camera, Jin et al. demonstrated compressed single-pixel hyperspectral imaging with a broad spectral range^23^. However, the trade-in of a single-pixel detector’s temporal bandwidth for spectral information makes the device incapable of measuring dynamic light^24^, such as time-of-flight signals.

To address these challenges, we develop a new category of cascaded compressed-sensing single-pixel cameras. Rather than relying on a single process to modulate a multidimensional light field, we decompose the compressed measurement into cascaded stages, sequentially reducing the dimensionality of the data from a high-D space to 0D. Such a measurement scheme allows us to exploit the compressibility of an object in multiple domains, which can lead to higher reconstructed image quality. Moreover, the accessibility to the intermediate image between adjacent measurement stages allows the mapping of high-D optical information to a low-D space through pure optical operations without sacrificing the temporal bandwidth of single-pixel detectors. We demonstrated our method in several formerly intractable applications, including enabling single-pixel full-waveform hyperspectral light detection and ranging (LIDAR) for the first time.

## Results

### Cascaded compressed measurement

**Fig. 1.**
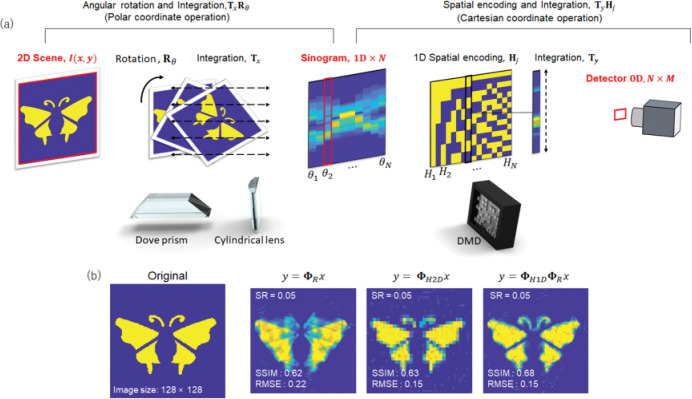
Principle of cascaded compressed sensing. **a.** Illustration of a single-pixel camera with cascaded compression. The 2D scene is rotated axially and integrated along the horizontal direction by a Dove prism and the cylindrical lens. The resultant multiple ID images at different rotation angles are composed into a sinogram of *en-face* parallel projections, similar to computed tomography. A DMD 3encodes a binary pattern onto the sinogram and integrates the signals along the horizontal direction. The single-pixel detector captures the resultant zero-dimensional data. **b**. Compressed imaging by using the en-face parallel projection method (*y* = **Φ**_*R*_*x*), 2D Hadamard-basis compression method (*y* = **Φ**_*H*2*D*_*x*), and cascaded compression method (*y* = **Φ**_*H*1*D*_**Φ**_*R*_*x*). *x* and *y* are the incident image and the measurement. **Φ**_*R*_, **Φ**_*H*2*D*_, and **Φ**_*H*1*D*_ are linear sensing matrices of *en-face* projection, 2D Hadamard encoding, and 1D Hadamard encoding, respectively. The sampling ratio is 0.05. SSIM structural similarity; RMSE, root mean squared error.

The core idea of our method is to transform light information in a high-D space to 0D through a cascaded process. For example, we show the optical realization of a two-stage system in Fig. 1, where we use two coupled subsystems to sequentially perform compressed measurements in the angular and spatial domains. In Stage I, we rotate the incident image using a Dove prism, followed by optically integrating the signals along the horizontal axis using a cylindrical lens. This transforms the input 2D image into a 1D line, essentially an *en-face* projection (or the radon transformation^25^) of the object^26^. Repeating this operation at different image rotation angles yields a sinogram like in computed tomography^27^. In Stage II, we take the 1D projection image from Stage I as the input and encode it with a Hadamard binary pattern using a spatial light modulator. The resultant light signals are then focused by a lens and spatially integrated at a single-pixel detector. The image formation model of the entire system can be formulated as

1 $$E_{j,\theta }=T_{y}V_{j}T_{x}R_{\theta }I(x,y).$$

Here **T**_*x*_ and **T**_*y*_ are integration operators along the horizontal and vertical axis, respectively. **V**_*j*_ is a 1D binary spatial encoding operator, and **V**_*j*_ = 1/2(**H**_1_ + **H**_*j*_), where **H**_*j*_ is the *j*^th^ Hadamard vector. **R**_*θ*_ is a rotating operator. *I*(*x*, *y*) denotes the incident 2D intensity image of *N* × *N* pixels. *E*_*j*_,_*θ*_ is the light intensity measured by the single-pixel detector. By choosing a subset of angular projections *θ* = *θ*_1_,*θ*_2_,…,*θ*_*r*_ and spatial samplings *j* = 1,2,…,*k*, we can capture the original 2D image compressively with a sampling ratio (SR) of (*r × k)*/*N*^2^.

Because optical operations are linear, we reconstruct the scene *I*(*x*, *y*) from the sparsely sampled measurement, **E**, by solving a linear inverse problem:

2 $${\mathrm{argmin}}_{\hat{I}}\|E-\Phi I{\|}_{2}^{2}+\lambda \phi (I),$$

where **E** is the vector representation of *E*_*j*_,_*θ*_ of size 1 × (*rk*), and Φ = **T**_*y*_**V**_*j*_**T**_*x*_**R**_*θ*_ is the sensing matrix that describes the linear relationship between the signal and the measurement. *λ* is the regularization parameter, and *ϕ*(*I*) is the regularization function, where we use *l*_1_-norm to force sparsity.

For high-quality image reconstruction, the system must satisfy two conditions: signal sparsity and incoherence of the measurement^28^. The signal is considered sparse if it can be represented in a few high-valued coefficients in an orthonormal basis as *I* = **Ψs**, where **Ψ** is the sparse basis matrix, and *s* is a sparse representation of the signal (i.e., *k* ≪*N*^2^, where *k* is the number of high-valued coefficients of *s*). To exploit the sparsity of the signal and recover the signal with high fidelity, the measurement matrix **Φ** must be incoherent to the sparse basis matrix **Ψ**. In other words, the correlation between any two columns in **Φ** and **Ψ** must be minimized^29^. In fact, the convex optimization algorithm of *l*_1_-norm minimization is also referred to as a basis-pursuit method^30^ that recovers the signal by finding its sparse representation.

However, without a priori knowledge of the signal, finding an optimal sensing matrix (or optimal measurement scheme) mutually incoherent to the sparse basis matrix is a demanding task^31^. For example, in Hadamard-basis compressed imaging, the image can be well reconstructed only when the signal is sparse in its original domain (space) but not in a transformed domain^32^. Also, the image reconstruction quality is susceptible to the choice of the subset of the Hadamard basis at a given sampling rate, especially under a highly under-sampled condition with noises^21^. In contrast, our method completely reshuffles the signals by a series of optical operations, including radon transformation (*en-face* parallel projection) and spatial projection, thereby facilitating the image reconstruction through the basis-pursuit *l*_1_-norm minimization (Fig. 1b).

### Numerical Simulations And Experimental Demonstrations

**Figure 2:**
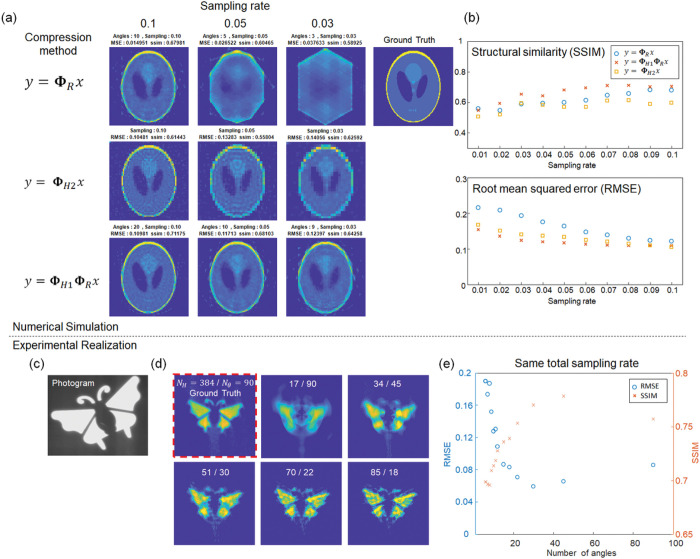
Image compression with three approaches: 2D Hadamard-basis compression method, *en-face* parallel projection method (sparse angle radon transformation), and cascaded compression method. **a** Reconstructed images with three different compression methods at a low sampling ratio. **b**. Root mean squared error (RMSE) and structural similarity (SSIM) of the reconstructed images. **c**. Photograph of a target scene. **d**. Experimental realization of the cascaded compressed measurement. **e**. RMSE and SSIM of the reconstructed scene. *N_H_* and *N_θ_* are the number of Hadmadard patterns and angles, respectively.

We performed numerical simulations to evaluate our method at a highly sub-Nyquist sampling rate. We chose a standard Shepp-Logan phantom image with 128×128 pixels as the test image (Fig. 2a) and simulated three compressed measurements: *en-face* parallel projection, 2D Hadamard basis compressed imaging, and cascaded compression. The target image was processed using the image formation model of each method, and white random gaussian noises were added to the image with a signal-to-noise ratio of 20 dB. We reconstructed the image using an iterative optimization algorithm (FISTA^33^ with a proximal operator of the *l*_1_-norm). To choose the subset of the Hadamard basis, we used total variation ordering^21^ because it typically yields a high-quality image given a low sampling rate, whereas no prior scene information is needed. For the *en-face* projection measurement, the projection angles are uniformly distributed in an angular range of [0, π] to maximize the information content. For a fair comparison, the sampling ratio is calculated as the sampling rate divided by the pixel count of the imaging eld of view. Specifically, the field of view is 128×128 = 16,384 pixels in the Hadamard basis compression method. In the other two methods, the field of view is computed as π × 64^2^=12,867, whereas it captures information within only the inscribed circular area of a squared scene.

The reconstructed images were evaluated based on two metrics: structural similarity (SSIM) and root mean squared error (RMSE). Figure 2a shows the reconstructed image from each method. The results indicate that the cascaded compression method provides a lower RMSE and a higher SSIM than the other two methods. In a highly under-sampled case (SR ≤ 0.05), the limited number of angles in the *en-face* projection method blurs the image along the angular direction. And the 2D Hadamard basis imaging method is plagued by pixelated artifacts because high-spatial frequency information is not addressable. Although a highly compressed inverse problem may be solved by using state-of-the-art optimization algorithms or deep-learning-based approaches^34^, it generally requires extensive computation or fine-tuning of the network with pre-acquired training datasets.

Next, we experimentally demonstrated single-pixel imaging using cascaded compression. A 2D image (Fig. 2c) was sequentially compressed by a two-stage system, and the light intensity was recorded at each dove prism rotation angle and Hadamard pattern. The reconstructed image is shown in Fig. 2d, where the image size is approximately π × r^2^ = π × 192^2^~116,000 pixels, and the samples acquired are 1,530. The corresponding sampling rate is SR = 0.013. Despite degraded image quality, the cascaded compression method provides a visually identifiable image with only 1.3% of the sampling rate. While the image quality improves as the overall sampling rate increases (Supplementary Information), the balance between the number of projection angles and the number of Hadamard patterns at a given sampling rate is critical to the image quality. Figure 2e illustrates the RMSE and SSIM of the reconstructed image compared to the densely sampled image. Noteworthily, there is no such an universal optimal measurement scheme^31^ because different images possess varied levels of sparsity. Our cascaded compression method provides an efficient sampling by enabling tunability on the sampling rate at each compression step.

### Single-pixel spectral imaging (x, y, λ)

**Figure 3:**
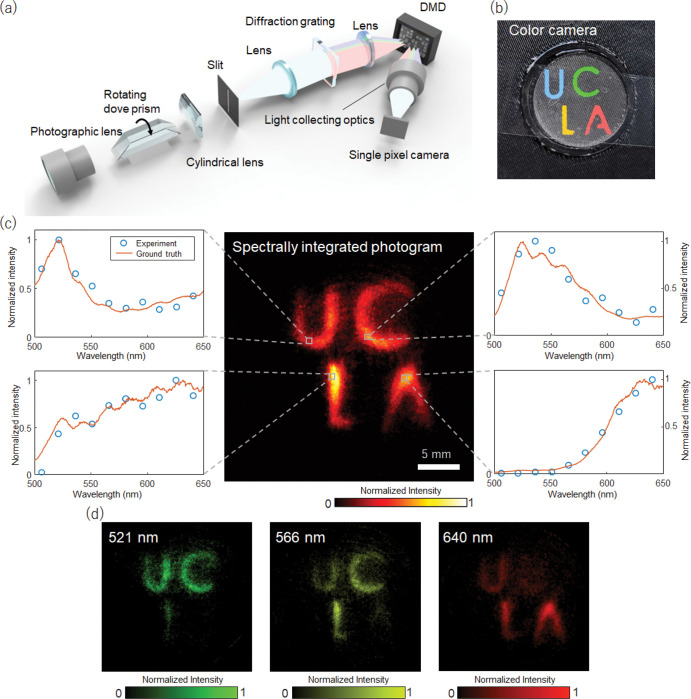
Single-pixel spectral imaging. **a**. System setup. After the *en-face* parallel projection, a 1D vertical line image was spectrally dispersed along the horizontal axis by a diffraction grating. **b**. Photogram of the target object. **c**. Spectrally integrated image. (Graphs) The spectral irradiance of the scene measured by the single-pixel camera and a benchmark fiber spectrometer. The spectrum was spatially integrated over 5×5 pixels. **d**. Visualization of the image at different wavelengths.

Compared with conventional single-stage systems, cascaded compressed sensing provides unique access to the intermediate image between adjacent measurement stages. Optical de-multiplexing of these images allows high-D light field information mapping to a low-D space, which can be further compressed in the subsequent stages. For example, in the two-stage system presented in Fig. 1a, the input image is first transformed into the 1D line through *en-face* projection. The reduced spatial dimension can be refilled with light information of higher dimensions, such as spectrum or polarization. We demonstrated this framework in hyperspectral imaging using a single-pixel camera (Fig. 3a). After Stage-I transformation, we spectrally dispersed the 1D line image using a diffraction grating, mapping the spectral information (500–650 nm, Supplementary Information) to the orthogonal spatial axis and restoring the spatial dimensions to 2D. The resultant image is then passed to the Stage II subsystem, where we use a digital micromirror device (DMD) for spatial encoding. We binned the DMD pixels along the spectral dispersion direction into ten super-pixels, each modulating a different light wavelength.

We tested our method on a colorful object fabricated using cellophane sheets and a diffuser (Fig. 3b). We back-illuminated the object with a halogen lamp and imaged the transmitted light. To capture a hyperspectral datacube of dimensions (384×384×10), we used a total of 45 image rotation angles and 192 orthonormal Hadamard patterns in Stage I and II compressed measurements, respectively, leading to an overall SR of 0.075 (the information content is within the inscribed circular area of the 384×384 pixels squared image). The reconstructed spectra and representative spectral channel images are shown in Fig. 3c and d, respectively. We also evaluated the accuracy of the spectral measurement by comparing the reconstructed spectra against the ground truth, which was obtained with a benchmark fiber spectrometer (STS-VIS-L-25-400-SMA, Ocean Optics). The mean squared error of the normalized spectral difference is 0.092.

The sampling rates of our cascaded compressed-sensing camera can be tuned in both spatial and spectral domains, allowing a tailored compressed measurement for a given scene. In the spatial domain, the sampling rate can be tuned by choosing the number of image rotation angles and Hadamard encoding patterns. While in the spectral domain, because the spectral information is directly mapped onto the horizontal axis of the DMD, we can acquire an arbitrary number of discrete spectral bands by modulating selective DMD pixel columns and choose a desired spectral resolution by binning the DMD pixels.

### Single-pixel spectral time-of-flight imaging (x, y, λ, t)

**Figure 4:**
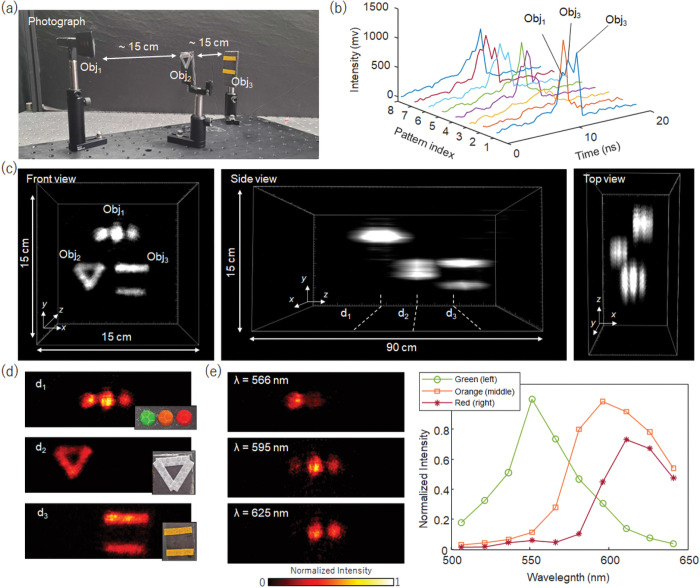
Single-pixel full-waveform spectral time-of-flight imaging. **a**. Photogram of the scene. The scene consists of three objects at different depths. **b**. Representative temporal waveforms. **c**. Reconstructed 3D scene visualized by the maximum intensity projection. **d**. Representative depth images. The insets show the ground truth photographs at the corresponding depths. **e**. Spectral images at depth d_1_ at representative wavelengths. **f**. Spectra of the colored circles at depth d_1_.

To measure high-D light eld information, conventional multidimensional optical imaging methods often trade in light information along another axis. In contrast, in a cascaded compressed-sensing camera, we employ an optical architecture that first reduces the spatial dimensionality of a light datacube, followed by transforming high-D light information to refill this spatial axis, thereby enabling a complete measurement of optical properties. Here, we demonstrate a four-dimensional (4D) (*x, y, λ, t*) spectral timeof-flight imaging by fully exploiting the bandwidth advantage of single-pixel detectors. We imaged a scene of colored objects positioned at different depths (Fig. 4a). The distance between the objects and the camera varied from 0.6 to 0.9 meters.

To cover a broad spectrum in the visible light range (500–650 nm), we illuminated the objects with a tunable pulsed laser (a femtosecond laser equipped with an optical parametric amplifier) stepwise, recorded the signals in each spectral band, and summed the raw data for processing. The spectral bandwidth of each laser pulse is approximately 20 nm, providing a sufficient spectral overlap between adjacent illuminations. The back-scattered light from the objects was captured by a two-stage compressed-sensing single-pixel camera (Fig. 3a) equipped with a high-speed APD (1.6 GHz bandwidth) and a digitizer (2.5 GHz digital sampling rate). We used a total of 45 image rotation angles and 192 Hadamard patterns, enabling the recording of 86,400 time-of-flight waveforms with a spatial SR of ~ 0.075 (Fig. 4b).

A 4D (*x, y, λ, t*) datacube with (384×384×10×15) voxels was reconstructed from the time-of-flight waveforms obtained and visualized in Fig. 4c. The temporal sampling rate of the digitizer is 2.5 GHz, leading to an axial sampling rate of ~ 6 cm (c*Δt*/2; *c*, speed of light in the air; *Δt*, time of flight), considering the round-trip travel time of the illuminating pulse. The 3D rendering recovers both the shape and depth of the object with high fidelity (Fig. 4c). The lateral field of view depends on the distance between the camera and the object because of a varying magnification, and it approximates 15 cm × 15 cm in the middle. The spectrally and temporally integrated 2D scene matches well with the reference image captured by a color camera (Fig. 4d). Figure 4e shows representative images at different wavelengths.

## Discussion

**Figure 5:**
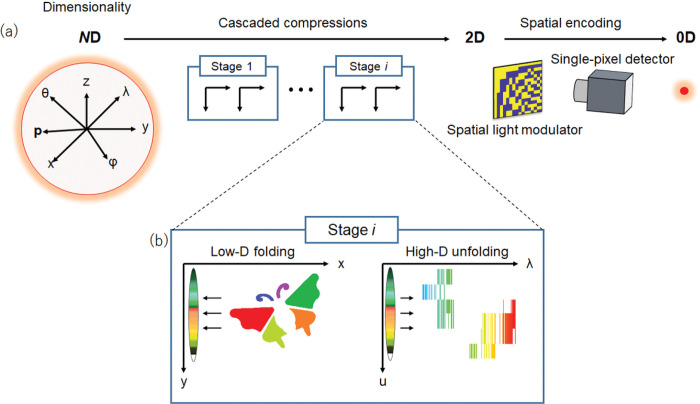
Conecptual framework of cascaded compressed imaging. **a**. High-D optical information is mapped into a low-D space by a series of optical operations. **B**.Low-D folding and high-D unfolding in a measurement stage. In this example, a 2D image is rst transformed into a 1D line during the low-D folding process, creating a blank spatial axis. The spectral information is then mapped to this axis during the high-D unfolding process.

The architecture of cascaded compressed imaging is scalable, allowing high-D optical information to be mapped to a low-D space stagewise. Figure 5 shows a generalized scheme with multiple compression stages, each consisting of a low-D folding and a high-D unfolding step. In the low-D folding step, the image is spatially compressed along the *x*-axis through optical transformation, creating a blank low-D axis. Next, in the unfolding step, we map the optical information along a high-D axis to this blank axis and measure the resultant image using computed tomography. Repeating these two steps at each compression stage allows measurement of an additional photon characteristic, and cascading multiple stages leads to a complete characterization. In the final stage, we further reduce the data dimensionality to 0D by modulating the image using a spatial light modulator and acquiring the spatially integrated signals using a single-pixel detector. Although we demonstrated only a two-stage system for (*x, y, λ*) and (*x, y, λ, t*) imaging, the cascaded compression framework can be expanded to measuring other photon characteristics, such as polarization or depth, by using a Wollaston prism^35^ or a volume hologram^36^, respectively, as a mapping device during high-D unfolding.

Cascaded compressed imaging is highly efficient in measuring a high-D light datacube. For conventional multidimensional imaging, acquiring optical information along an extra dimension often yields a significant dataload, posing challenges in data transfer, storage, and processing. The cascaded compression alleviates this problem by mapping the optical information along the axis of interest to a low-D spatial axis in a compressed manner rather than measuring the complete information. Such a measurement scheme is particularly suitable for multidimensional imaging because it exploits the sparsity of natural scenes in a high-D space that cannot be commonly found in a low-D space. For example, in 4D full-waveform hyperspectral time-of-flight imaging, the spectrally resolved image at each time instant of the reflected time-of-flight signal is far simpler than a conventional color photograph, where the light signals are temporally and spectrally integrated in the same image. As a result, the optical information along the unfolded spectral and temporal axes is intrinsically sparse, and it can be measured at a much lower rate than the Nyquist sampling condition. Given this prior knowledge on signal sparsity, we can faithfully recover a high-D datacube with only a small fraction of information recorded. The same principle is also applicable to acquiring other light datacube dimensions. For example, we demonstrated 4D(*x, y, u, v*) compressed light field imaging and presented the results in Supplementary Fig. 1.

Lastly, the competitive advantage of cascaded compressed imaging over conventional DMD-based single-pixel cameras exists not only in high-D imaging but also in low-D imaging. For example, when capturing a 2D image with conventional Hadamard encoding, the limited onboard memory size of DMDs usually leads to insufficient samplings for high-resolution imaging. As a result, the image size of most single-pixel cameras using a DMD is limited to a few tens of thousands of pixels, far fewer than the resolution of a conventional digital camera. In contrast, by decomposing the measurement into two stages, we loaded only 1,920 patterns on the DMD and captured 86,400 samples. Conventional DMD-based single-pixel cameras cannot achieve this because the number of samples acquired exceeds the modulation capability of the DMD (64 Gbit, maximum 55,924 patterns).

In conclusion, we developed a new category of compressed-sensing cameras capable of capturing a multidimensional optical eld using a single-pixel detector. By mapping high-D information to a low-D space through a cascaded process, we enable a highly efficient measurement of a light datacube with rich information contents. We expect our method will find broad multidimensional imaging applications that use single-pixel detectors.

## Methods

### Experimental setup

We collected the image using a dove prism (PS992M, Thorlabs) and cylindrical lens (*f* = 100 mm) with an optical power along the vertical direction (Fig. 3a). The dove prism was mounted on a high-speed rotary stage (ACR32UT, IntelLiDrives). The image was then projected onto a DMD (V-650L, Vialux) through a 4-*f* telescopic imaging system with a unity magnification (*f* = 75 mm). In practice, the DMD was mounted on a stage inclined at 45 degrees so that the reflection beam direction is parallel to the optical table. A diffraction grating (GT-25-03, 300 grooves/mm) was positioned at the Fourier plane of the imaging system to disperse the light along the horizontal direction. We can encode the image with a desired binary pattern by modulating the micromirrors on the DMD.

The light-collecting optics after the DMD consist of a 4-*f* telescoping imaging system with a diffraction grating at the Fourier plane, which un-disperse the beam. The focal length of the lenses and the groove density of the diffraction grating are the same but with a reversed dispersion direction. After passing the light-collecting optics, the light is directed to the single-pixel detector (APD210, 1.6 GHz, MeloSystems) and digitized by the high-speed digitizer (6426E, 2.5 GHz, Picoscope) for processing.

For time-of-flight imaging, we use a Ti:Sapphire femtosecond laser (Astrella-F-1K, 808 nm, 100 fs pulse width, Coherent) as an illumination source. An optical parametric amplifier (TOPAS-Prime, Light Conversion) further converts the laser wavelength to the visible light range. The trigger-out from the mode-locked laser was used as a reference for accurate timing for the digitizer.

### System Calibration

A lateral shift of the image at the DMD plane often accompanies the rotation of the Dove prism in our system due to misalignment of the system’s optical axis with respect to the center of rotation of the Dove prism. The deviation was corrected computationally by introducing a lateral shifting operator into the linear forward model.

To determine the amount of the lateral shift of the image varied with the Dove prism rotation angle, we positioned a pinhole at the optic axis of the system, illuminated it with a narrow band laser beam (λ = 532 nm), and captured the image using a conventional Hadamard basis single-pixel imaging configuration. The exact spatial location of the mapped laser beam was recorded, and the deviation from the center of the DMD was calculated at different rotation angles of the Dove prism. The corresponding lateral shifting operator was compensated when reconstructing the image.

We calibrated the relation between the spectrum of the incident light and the spatial location of the light mapped onto the DMD. We positioned a pinhole at the object plane of the system and illuminated it with 550 nm, 590 nm, and 630 nm wavelength light. On the DMD, the light signals are spectrally dispersed along the horizontal axis and sampled by 360 horizontal pixels. Along the vertical direction, the DMD pixels were grouped into 72 stripes, each having a width of five pixels. We sequentially turned on each stripe on the DMD and recorded the corresponding intensities on the single-pixel detector, similar to a push-broom scanner. Horizontal locations of the 550 nm, 590 nm, and 630 nm light on the DMD were founded by interpolations between the horizontal pixel index of the stripes and the measured intensity. In hyperspectral imaging, a total of 360 horizontal pixels of the DMD was used to map the spectral information of the scene within the range of approximately 500 nm to 650 nm (Supplementary Fig. 3).

The spectral radiance response was calibrated using a white lamp and a benchmark spectrometer (STS-VIS-L-25-400-SMA, Ocean Optics). An iris was positioned at the object plane of the system and illuminated by a white lamp. The light was dispersed along the horizontal direction and mapped onto the DMD. The DMD was divided into ten vertical stripes with a horizontal pixel size of 36 pixels. The number of spectral channels for calibration was the same as the number of spectral channels used throughout this research. The corresponding spectral range for each channel (stripe) was approximately 15 nm, and the spectral range was approximately 500 nm to 650 nm. We sequentially turned on the vertical stripe images on the DMD and recorded the intensity value by the single-pixel detector for each stripe. The spectral response of the system was normalized by comparing it with the spectrum measured by a benchmark spectrometer (Supplementary Fig. 3).

### Image Reconstruction

To reconstruct the image from the measurement, we solved the linear inverse problem of Eq. (2). We adopted a fast iterative shrinkage-thresholding algorithm^33^ with a proximal operator of the *l*_1_-norm^27^ for the regularization, which lessens the computational cost compared to the use of state-of-art regularizers such as total variation and wavelet transformations. The hyperparameter *μ* was chosen between 0.05 to 0.5, which does not significantly affect the image reconstruction fidelity.

## Data Availability

The data that support the plots within this paper and other findings of this study are available from the corresponding author upon reasonable request.
